# Deletion of *Ripk3* Prevents Motor Neuron Death *In Vitro* but not *In Vivo*

**DOI:** 10.1523/ENEURO.0308-18.2018

**Published:** 2019-02-19

**Authors:** Georgia Dermentzaki, Kristin A. Politi, Lei Lu, Vartika Mishra, Eduardo J. Pérez-Torres, Alexander A. Sosunov, Guy M. McKhann, Francesco Lotti, Neil A. Shneider, Serge Przedborski

**Affiliations:** 1 Department of Pathology and Cell Biology, Columbia University, New York, NY 10032; 2 Department of Neurology, Columbia University, New York, NY 10032; 3 Department of Neurological Surgery, Columbia University, New York, NY 10032; 4 Center for Motor Neuron Biology and Disease, Columbia University, New York, NY 10032

**Keywords:** ALS, mice, motor neuron, necroptosis, neurodegeneration, Ripk3

## Abstract

Increasing evidence suggests that necroptosis, a form of programmed cell death (PCD), contributes to neurodegeneration in several disorders, including ALS. Supporting this view, investigations in both *in vitro* and *in vivo* models of ALS have implicated key molecular determinants of necroptosis in the death of spinal motor neurons (MNs). Consistent with a pathogenic role of necroptosis in ALS, we showed increased mRNA levels for the three main necroptosis effectors *Ripk1*, *Ripk3*, and *Mlkl* in the spinal cord of mutant superoxide dismutase-1 (SOD1^G93A^) transgenic mice (Tg), an established model of ALS. In addition, protein levels of receptor-interacting protein kinase 1 (RIPK1; but not of RIPK3, MLKL or activated MLKL) were elevated in spinal cord extracts from these Tg SOD1^G93A^ mice. In postmortem motor cortex samples from sporadic and familial ALS patients, no change in protein levels of RIPK1 were detected. Silencing of *Ripk3* in cultured MNs protected them from toxicity associated with SOD1^G93A^ astrocytes. However, constitutive deletion of *Ripk3* in Tg SOD1^G93A^ mice failed to provide behavioral or neuropathological improvement, demonstrating no similar benefit of *Ripk3* silencing *in vivo*. Lastly, we detected no genotype-specific myelin decompaction, proposed to be a proxy of necroptosis in ALS, in either Tg SOD1^G93A^ or *Optineurin* knock-out mice, another ALS mouse model. These findings argue against a role for RIPK3 in Tg SOD1^G93A^-induced neurodegeneration and call for further preclinical investigations to determine if necroptosis plays a critical role in the pathogenesis of ALS.

## Significance Statement

Studies in models of ALS suggest an instrumental role for necroptosis in this currently incurable disease. Given the availability of a growing number of small molecules that inhibit key determinants of necroptosis, implication of this form of programmed cell death (PCD) in ALS may open new therapeutic avenues. We found that despite changes in the expression of key necroptosis determinants, targeting receptor-interacting protein kinase 3 (RIPK3) prevents motor neuron (MN) death *in vitro* but not *in vivo*. These results challenge the suggestion that targeting RIPK3 to abrogate necroptosis will provide therapeutic benefit in ALS.

## Introduction

ALS is an adult-onset paralytic disorder that is characterized by the loss of upper and lower motor neurons (MNs). Neurodegeneration results in muscle weakness leading to difficulty in moving, speaking, swallowing and eventually breathing. The disease progresses rapidly with a mean survival of three to five years after diagnosis. To date, riluzole and edaravone are the only two Food and Drug Administration-approved ALS drugs, each of which has only marginal therapeutic benefit (Miller et al., 2007; Abe et al., 2014). Given these facts, it is estimated that more than two in every 100,000 Americans will die from ALS, unless a more effective treatment is found. Advances in our knowledge of the mechanisms involved in disease pathogenesis will undoubtedly lead to the development of novel therapies for ALS.

Inhibition or deletion of the death agonist Bax prevents MN degeneration in both cell and animal models of ALS (Gould et al., 2006; Nagai et al., 2007; Re et al., 2014). In contrast, selective antagonists of executioner caspases and the pan-caspase inhibitor zVAD do not attenuate MN loss in cell culture models of familial and sporadic ALS (Nagai et al., 2007; Re et al., 2014) and are only mildly protective in Tg SOD1^G93A^ ALS mice (Li et al., 2000). Furthermore, while some authors have shown evidence of caspase activation in ALS mouse models and even in human tissue (Friedlander et al., 1996; Martin, 1999; Li et al., 2000; Pasinelli et al., 2000; Vukosavic et al., 2000; Inoue et al., 2003), others have failed to detect clear signs of apoptosis in degenerating MNs in mouse models of ALS (Migheli et al., 1999). Collectively, these findings argue that a caspase-independent form of programmed cell death (PCD) may underlie neurodegeneration in ALS. Previous studies focusing on the molecular basis of MN death in *in vitro* models (Nagai et al., 2007; Re et al., 2014; Ikiz et al., 2015) have demonstrated that pharmacological or genetic targeting of key determinants of necroptosis, a molecularly-controlled form of necrosis (Grootjans et al., 2017) prevented MN death (Re et al., 2014). Further evidence that necroptosis may be involved in ALS neurodegeneration comes from *in vivo* studies in which the administration of the small molecule Nec-1s, a kinase antagonist of the receptor-interacting protein kinase 1 (RIPK1), or deletion of RIPK3, two key determinants of necroptosis (Grootjans et al., 2017), was shown to delay the onset of the ALS phenotype in Tg SOD1^G93A^ mice (Ito et al., 2016). These authors (Ito et al., 2016) also showed that inhibiting necroptosis rescued axonal myelination defects in both Tg SOD1^G93A^ mice and in mice deficient in optineurin (*Optn^−/−^*), another ALS mouse model.

Given the potential therapeutic value of targeting necroptosis in ALS, the goals of the present study were first, to confirm the involvement of this form of PCD in ALS neurodegeneration by quantifying transcript and protein levels of core necroptosis determinants in affected regions of the central nervous system. Second, since previous studies only reported on the effects of targeting necroptosis on the onset of motor dysfunctions (Ito et al., 2016), we sought to characterize the benefits of an anti-necroptosis strategy on motor performance and survival in ALS mice to provide preclinical evidence to support this approach to treat ALS patients. Third, since Ito et al. (2016) invoke activation of necroptosis in both oligodendrocytes and microglia, but not in spinal MNs, while *in vitro* models suggest the activation of necroptosis within MNs, we aimed here to resolve this discrepancy by elucidating the cellular site of action of necroptosis in ALS.

## Materials and Methods

### Animals

All experimental procedures followed the National Institutes of Health Guide for Care and Use of Laboratory Animals (National Research Council, 2011). All animal procedures were approved and performed in accordance with the institutional animal care and use committee’s policies at Columbia University. A total of ∼75 male and female mice were group-housed in polycarbonate cages with corncob bedding; they were maintained in a humidity- and temperature-controlled vivarium (20–22°C) on a 12/12 h light/dark schedule. Animals had access *ad libitum* to food and water except during behavioral testing.

*Ripk3^−/−^* and *Ripk3*^+/−^ female mice (C57Bl/6N; Newton et al., 2004), kindly provided by Genentech (MTA: OM-213900), were bred with male B6.Cg-Tg(SOD1*G93A)1Gur/J (https://www.jax.org/search?q=004435). Tg SOD1^G93A^; *Ripk3^+/+^* (*n* = 20) and Tg SOD1^G93A^;*Ripk3^−/−^* (*n* = 23) mice (male and female) were assessed for survival, onset of disease, motor function via grip strength tests, MN number in the lumbar spinal cord, and innervation of the neuromuscular junction (NMJ) at the tibialis anterior (TA) muscle. In addition, B6SJL-Tg(SOD1*G93A)1Gur/J (Tg SOD1^G93A^) (https://www.jax.org/strain/002726; *n* = 6), B6SJL-Tg(SOD1)2Gur/J (SOD1^WT^) (https://www.jax.org/strain/002297; *n* = 6) mice, and control littermates (*n* = 6), were also used for assessing mRNA and protein expression levels of RIPK1, RIPK3, MLKL and p-MLKL. For *Ripk3*, genotyping was performed using a mix of primers: 5′-AGAAGATGCAGCAGCCTCAGCT-3′, 5′-ACGGACCCAGGCTGACTTATCTC-3′ and 5′-GGCACGTGCACAGGAAATAGC-3′ for both wild-type (WT) and *Ripk3* knock-out. For mutant SOD1^G93A^, human transgene genotyping was performed using protocols and primer sequences obtained from The Jackson Laboratory.

Optn^tm1a(EUCOMM)Wtsi^ ES cells were purchased from The Jackson Laboratories (http://www.informatics.jax.org/allele/MGI:4432769). Using standard protocol, ES cells were then injected into the blastocoel cavity of 3.5-d-old mouse blastocysts which were transferred surgically to the uterine horns of appropriately timed pseudo-pregnant recipient females which gestated normally (Conner, 2001). Chimeric pups were then genotyped and used for further crossing to generate *Optn* knock-out mice. *Optn* mutation details: the L1L2_Bact_P cassette was inserted at position 5053776 of chromosome 2 upstream of the critical exon(s) (Build GRCm38). The cassette was composed of a FRT site followed by a lacZ sequence and a loxP site. This first loxP site was followed by neomycin under the control of the human beta-actin promoter, SV40 poly A, a second FRT site and a second loxP site. A third loxP site was inserted downstream of the targeted exon at position 5052564. LoxP sites thus flanked the critical exon. A “conditional ready” (floxed) allele was created by flp recombinase expression in mice carrying this allele. Subsequent UBC-cre expression resulted in a knock-out mouse. After one more cross, Optn knock-out homozygous mice were generated and used for the experiments; littermates were used as controls. For *Optn*, genotyping was performed using primer sets: forward: 5′-GCAGGGGCATTCTAAGTTCA-3′, reverse: 3'-TCCCTGCAAATTCCTTTCTG-5' and forward: 5′-TCTGAACCCCAAACAGAAGC-3′, reverse: 5′-GCTCTTCCTTCAGCCTCTCA-3′, for WT and knock-out Optn, respectively. Optn^+/+^ (*n* = 3) and Optn^−/−^ (*n* = 3) were assessed for MN number in the lumbar spinal cord and for innervation of the NMJ at the TA muscle.

### Cell cultures

L-929 cells (CVCL_0462) were cultured in DMEM (12430-054; Gibco), 10% horse serum (HS; 26050-070; Gibco), 0.5 mM L-glutamine (25030-081; Gibco), and 1% penicillin/streptomycin (P/S; 15140-122; Gibco).

For mixed-lineage kinase domain-like (Mlkl) silencing, L929 cells were infected with lentivirus-containing shRNA against Mlkl (360819; Sigma Mission) at a multiplicity of infection (MOI) 100. Four days later, cells were harvested and protein extraction was performed to assess knock-down efficiency.

For inducing MLKL phosphorylation (p-MLKL), L929 cells were treated with 30-ng/ml human recombinant TNF-α (cyt-223-b; Prospec), 100 nM smac mimetic (TL-32711; A1901; Active Biochem), and 20 μM zVAD-fmk (V116; Sigma; TSZ) for 1 h, and then protein was extracted.

NIH 3T3 cells (CVCL_0594), were cultured in DMEM (12430-054; Gibco), 10% fetal bovine serum (FBS; A31604-02; Gibco), 0.5 mM L-glutamine (25030-081; Gibco), and 1% P/S (15140-122; Gibco). This cell line expresses high levels of MLKL and was used as positive control for testing anti-MLKL antibody.

### Primary neuron-astrocyte co-cultures

Primary glia cultures were prepared from postnatal day 1 (P1) non-transgenic (NTg) or Tg SOD1^G93A^ mouse cortex. Briefly, cerebral cortices (*n* = 1 per 75-cm^2^ flask) were dissociated with an 18-G needle (305196; BD PrecisionGlide) and cell suspensions were plated in medium: DMEM (12430-054; Gibco), 10% FBS (A31604-02; Gibco), and 1% P/S (15140-122; Gibco) in 75-cm^2^ flasks. To eliminate residual microglia, two-week-old cultures were agitated (200 rpm for 6 h), astrocytes were detached by 0.05% trypsin-EDTA (25300-054; Gibco) and plated onto 0.01% poly-D-lysine- (P1274; Sigma) and 15 μg/ml laminin-coated (23017-015; Invitrogen) coverslips in 24-well plates at a density of 80,000 cells per coverslip.

Primary MN (PMN) cultures were prepared from embryonic day 12.5 (E12.5) *Ripk3^+/+^* or *Ripk3^−/−^* mouse spinal cords. Briefly, spinal cords (*n* = 5–6) were incubated in 0.025% trypsin (15090-046; Gibco) and 1-mg/ml DNase (LK003170; Worthington) for 10 min at 37°C. Next, cells were mechanically dissociated by pipetting through a 1-ml pipette tip, placed on a 4% BSA (A9418; Sigma) cushion, and centrifuged at 300 × *g* for 5 min. Cell pellet was resuspended in PMN media: neurobasal medium (ME120079L2; Gibco), 2% B27 supplement (17504-044; Gibco), 2% HS (26050-070; Gibco), 0.5 mM L-glutamine (25030-081; Gibco), 25 μM β-mercaptoethanol (ES-007-E; Millipore), and 1% P/S (15140-122; Gibco), supplemented with 1 ng/ml BDNF (450-02-10UG; ReproTech), 0.5 ng/ml GDNF (450-10-10UG; PeproTech), 10 ng/ml CNTF (450-13-20UG; ReproTech), and plated on top of astrocyte monolayers (obtained as indicated above) at a density of 35,000 cells per well. Co-cultures were kept for 7 d, fixed with 4% paraformaldehyde (PFA), and stained with the anti-neurofilament H non-phosphorylated (SMI32) antibody (1/500; AB_2043449), a reliable marker of MNs. Number of MNs was assessed by counting SMI32-immunolabeled cells under the microscope.

### RNA extraction-cDNA synthesis-qPCR

Total RNA was extracted from lumbar spinal cords (L1-L5 segment) using TRI reagent (T9424; Sigma) following the manufacturer’s protocol. DNase treatment was performed using rDNAseI (AM2235; Ambion) to remove any remaining DNA, followed by phenol chloroform extraction to ensure high RNA quality. RNA concentration was determined spectrophotometrically at 260 nm. Quality of the RNA was determined by the 260/280 and 260/230 ratio. cDNA was generated with the RevertAid First Strand cDNA Synthesis kit (K1691; ThermoFisher) following manufacturer’s protocol. For the reaction, we used 1 μg of RNA primed with both random hexamers and oligo(dT) primers. A three-step real-time qPCR was conducted with the Realplex 4 Mastercycler PCR System (Eppendorf) using SYBR Green dye (4367659; ThermoFisher).

The following primers were used for the real-time qPCR: *Ripk1* F': 5′-GGAGCCCTATGAGAATGTCATCTG-3′; *Ripk1* R': 5′-TCCTCTACATTTGGCCTGTTCC-3′, *Ripk3* F': 5′-ATCCTTCCAGGACTGCGAACCAAA-3′, *Ripk3* R': 5′-GCAGCATCTACCTT GTCCTTTACCAG-3′, *Mlkl* F': 5′-CCTGAAGCAATGCTCACTAAAACCC-3′, *Mlkl* R': 5′-CACGGAGGTCCAAGATGTTCCTTT-3′, *Gapdh* F': AACTCCCACTCTTCCACCTTCGAT-3′, 5'-, *Gapdh* R': 5′-CCCTGTTGCTGTAGCCGTATTCAT-3′. Fold difference was calculated as 2^-ΔΔCt^.

### Lentiviral-mediated gene silencing in mouse PMN cultures

Primary neuronal cultures from E12.5 mouse embryos were prepared as described above and diluted at a final concentration of 1 × 10^5^ cells/ml. Next and before plating, cells were infected with pLKO.1-puro plasmids (Sigma Mission); TRCN0000022468 clone (targeting Ripk1); or the SHC002H (scrambled) at a MOI of 20 following the spinoculation protocol (https://www.sigmaaldrich.com/life-science/functional-genomics-and-rnai/shrna/learning-center/spinoculation-protocol.html). Cells were then centrifuged at 800 × *g* for 30 min at room temperature, resuspended in fresh PMN media, and seeded at a density of 120,000 cells on 0.01% poly-D-lysine-coated (P1274; Sigma) and 15 μg/ml laminin-coated (23017-015; Invitrogen) 24-well plates. Four days after infection, cells were harvested and assessed for RIPK1 protein expression by Western blotting. For MNs, infections were performed in the absence of hexadimethrine bromide and puromycin, since the addition of these factors did not enhance the knock-down efficiency in these non-dividing cells.

### Western blotting

Mice were anaesthetized with ketamine-xylazine and perfused intracardially with 0.1 M ice-cold PBS (4190136; ThermoFisher) for 4 min at 10 ml/min. Spinal cords were removed, frozen on dry ice, and stored at –80°C in preweighed tubes. On the day of the lysis, cords were thawed on ice and weighed. Lysis buffer was added at a ratio of 1 ml/100 mg of tissue.

Lumbar spinal cords (L1-L5 segment) were homogenized either in RIPA [150 mM NaCl, 25 mM Tris (pH 8.0), 1% Triton X-100, 0.5% sodium deoxycholate, 1 mM ETDA, and 0.1% SDS] or in 6 M urea lysis buffer [50 mM Tris-HCl (pH 7.5), 150 mM NaCl, 1% NP-40, 1 mM EGTA, 50 mM NaF, 10 mM β-glycerophosphate, 5 mM sodium pyrophosphate, 1 mM sodium orthovanadate, 1 mM benzamidine, 1 mM PMSF, 5 mM N-ethylmaleimide, and 1 mM DTT] containing protease (11836153001; Roche) and phosphatase inhibitor cocktail (04906845001; Sigma).

For RIPA lysis buffer, tissue was first homogenized then sonicated (2.5 Hz, 10 s, two times), and clear lysate was isolated following centrifugation at 13,000 rpm for 15 min. For the 6 M urea lysis buffer, tissue was first homogenized in buffer containing all of the ingredients except 6 M urea, then sonicated (as for RIPA) and incubated for 1 h at 4°C with rotation. After centrifugation at 15,000 rpm for 20 min, the supernatant was removed and the pellet was washed twice with PBS (14190136; ThermoFisher). A total of 6 M urea-containing buffer was added and the pellet was resuspended, sonicated, then re-incubated at 4°C with rotation for 1 h. Protein concentrations were determined using the Bradford Quick Start assay (500-0205; Bio-Rad) and DC Protein Assay (Bio-Rad) for the RIPA and the 6 M urea lysis buffer, respectively. Approximately 50 μg of protein in the lysates was mixed with 5× Laemmli buffer (Tris-HCl pH 6.8, 10% SDS, 25% glycerol, 5% β-mercaptoethanol, and 0.05% bromophenol blue) to a final concentration of 1× before running on 4–12% Bis-Tris precast gels (NP0341BOX; Life Technologies). Following transfer to a nitrocellulose membrane, the blots were probed with antibodies directed against: RIPK1 (1:1000; AB_394014), RIPK3 (1:1000; AB_2722663), MLKL (1:1000; AB_11134649), p-MLKL (1:1000; AB_2687465) overnight at 4°C. β-ACTIN (1:40,000; AB_476744) and GAPDH (1:10,000; AB_1080976) were used as loading control. Blots were probed with either fluorescent (Li-Cor buffer 1:20,000 IR-700 dye conjugated; mouse, rabbit) or HRP-conjugated (mouse, rabbit) secondary antibodies (1:3000; NXA931: mouse, NA93AV: rabbit, GE Healthcare UK Ltd). Fluorescent imaging was performed with the Li-Cor Odyssey Imaging system. Chemiluminescent imaging was performed with Supersignal West Pico Chemiluminescent Substrate (34080; ThermoScientific) and visualized with X-Ray Films (Medilink Imaging). Following scanning of the images, ImageJ analyzer was used to quantify the optical density of the bands. In all cases, the levels of the proteins of interest were normalized to those of β-ACTIN or GAPDH for quantification and statistical analysis.

For *Optn^+/+^*, *Optn^+/−^*, and *Optn^−/−^* mice, spinal cord tissues were homogenized and proteins were extracted with LDS buffer (NP008; Invitrogen) and processed for Western blotting as described above. Antibodies against optineurin were AB_2156554 (1:1000) and against GAPDH were AB_1078991 (1:10,000).

For human brain samples, Brodmann’s area 4, which contains the motor cortex, homogenates were prepared in lysis buffer [0.1 M MES (pH 7), 1 mM EDTA, 0.5 mM MgSO_4_, 1 M sucrose, 50 mM nethylmaleimide,1 mM NaF, 1 mM Na_3_VO_4_, 1 mM PMSF, and 10 μg/ml each of aprotinin, leupeptin, and pepstatin] and processed for Western blotting as described above. We used two RIPK1 antibodies (AB_394014 and AB_397831).

### Transmission electron microscopy (TEM)

TEM was performed as described previously (Sosunov et al., 2017). Briefly, mice were anaesthetized with isoflurane before intracardiac perfusion with ice-cold PBS followed by ice-cold 2.5% glutaraldehyde + 2% PFA in 0.1 M phosphate buffer (pH 7.4). Spinal cords with ventral and dorsal roots at the lumbar level (L1-L4) were removed under a dissecting microscope and kept in the fixative for 12–16 h (4°C). After postfixation in 2% osmium tetroxide in 0.2 PBS (2 h at 4°C) and dehydration, small pieces of tissue were embedded in Epon-Araldite (Electron Microscopy Sciences). Semi-thin sections stained with toluidine blue were used for orientation. Ultrathin sections were cut with Reichert Ultracut E, contrasted with uranyl acetate and lead citrate, and examined with a JEOL 1200 electron microscope. For each genotype, three to four mice were used.

### Mouse behavioral tests

The loaded grid test was performed as previously described by Barnéoud et al. (1997). Briefly, the mouse was suspended by the tail and allowed to grip a series of grids with increasing weights (10, 20, 30, 40 g) and a behavioral score was calculated as follows: score = [Σ(tw × W)]/body weight, where tw corresponds to the maximum time the mouse was able to hold the grid at weight W. Body weight is the weight of the animal at the time of the test. A maximum period of 30 s was allowed for each weight. The best of three trials was recorded with at least 15-s resting period between each trial of the same weight and at least 30-s resting period between each new weight. Inverted grid or “wire-hang” test was performed by first allowing the mouse to grip a grid and then inverting the grid, thus allowing the mouse to hang. Score was recorded as maximum hold time up to 60 s, as the best of three trials with at least 3-min resting period between each trial. This test was always performed on a day other than the loaded grid test day. Animals were tested once per week at roughly the same time of day throughout the trial.

### Quantification of innervated NMJs of the TA muscle

Mice were anaesthetized with ketamine-xylazine and perfused intracardially with 0.1 M ice-cold PBS (4190136; ThermoFisher) for 4 min at 10 ml/min followed by ice-cold 4% PFA in 0.1 M PBS for 8 min at 10 ml/min. TA muscle was dissected out and incubated in PFA overnight at 4°C, then transferred to 30% sucrose in 0.12 M phosphate buffer at 4°C for at least 48 h. The TA muscles were subsequently placed in OCT (Tissue-Tek) in molds on dry ice and stored at –80°C until cryosectioning. Cryosections of TA (20 µm) were cut and slide-mounted. NMJs were immunolabeled for presynaptic marker VaChT (1:2000; AB_261875) or synaptophysin (Syn; 1: 500; AB_86671) or postsynaptic nicotinic acetylcholine receptors using fluorescent-conjugated BTX (1:200 α-bungarotoxin conjugated to Alexa Fluor 594; Invitrogen). NMJ innervation was quantified by identifying BTX-positive NMJs (red) and determining the extent of co-localization with VAChT (green). Full innervation is considered greater than a 70% overlap of BTX with VaChT, partial at 30–70% overlap, and denervation at 0–30% overlap. Imaging was performed with a fluorescence microscope at 10× magnification. At least 100 NMJs on 20-µm TA sections were imaged and counted for each mouse.

### Quantification of MNs in the mouse spinal cord

Mice were anaesthetized with ketamine-xylazine and perfused intracardially as described above. Spinal cords were removed and postfixed in PFA overnight at 4°C. The L4-L5 segment, identified by its ventral roots, was carefully dissected out and incubated at 4°C in 10% sucrose in 0.12 M phosphate buffer for at least 48 h. The spinal cord was then incubated in 7.5% gelatin/10% sucrose solution at 37°C for 1.5 h followed by embedding in a gelatin/sucrose solution in a plastic mold at 4°C until firm for not >1 d. Spinal cords in gelatin were then cut into blocks and flash frozen for 45 s in 2-methylbutane, dry ice cooled to –60°C and stored at –80°C until sectioning; 20-µm sections were cut on a cryostat and every other section was collected for immunostaining with the MN-specific marker ChAT (1:100; AB_2079751). An entire set of minimum 100 sections (20-µm thickness) from L4-L5 was counted, and the average number of MNs per ventral horn was obtained. A neuron would be counted if the nucleus, as identified by a DAPI counterstain, was present in the optical plane.

### Statistics

All datasets are expressed as mean ± SEM. Differences between variables were analyzed by Student’s *t* test (with Bonferroni correction to correct for multiple comparisons, whenever indicated) and among variables by one-way ANOVA or two-way ANOVA followed by Tukey’s or Newman–Keuls *post hoc* tests, respectively. RIPK1 protein levels and clinical information were tested for correlation by linear regression. Survival and onset were analyzed via log-rank Mantel–Cox test. All statistical analyses were run on SigmaPlot 12.0 (Systat Software, Inc.), and the null hypothesis was rejected at the 0.05 level, unless corrected for multiple comparisons.

## Results

### Expression of key molecular determinants of necroptosis in Tg SOD1^G93A^ mice

**Figure 1.**
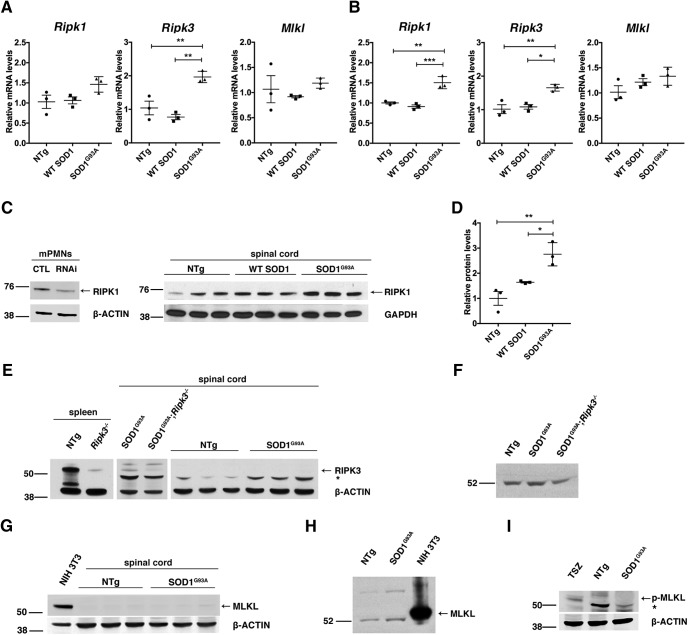
Upregulation of core necroptosis components in the spinal cord of symptomatic Tg *SOD1^G93A^*mice. Lumbar spinal cords, from 12- and 15-week-old Tg SOD1^G93A^, WT SOD1, and NTg mice, were isolated and processed for mRNA and protein (RIPA or urea extraction) expression of RIPK1, RIPK3, MLKL, and p-MLKL. ***A***, Quantification of *Ripk1*, *Ripk3*, and *Mlkl* mRNA from 12-week-old mice*. Gapdh*: housekeeping gene. A significant increase was detected for *Ripk3* in Tg *SOD1^G93A^* compared to Tg SOD1^WT^ (*p* = 0.0021) and NTg (*p* = 0.0076) at 12 weeks. ***B***, Quantification of *Ripk1*, *Ripk3*, and *Mlkl* mRNA in spinal cords of 15-week-old mice*. Gapdh*: housekeeping gene. A significant increase was detected for *Ripk1* (*p* = 0.0009, vs Tg SOD1^WT^; *p* = 0.0020, vs NTg) and *Ripk3* (*p* = 0.0115; vs Tg SOD1^WT^, *p* = 0.0067; vs NTg) but not for *Mlkl* in Tg SOD1^G93A^ compared to Tg SOD1^WT^ and NTg mice at 15 weeks. ***C***, Western blotting (RIPA) for RIPK1 in spinal cord of NTg, Tg SOD1^WT^, and Tg SOD1^G93A^ 15-week-old mice. β-ACTIN, GAPDH: loading control. Specificity of the RIPK1 band was confirmed following downregulation of RIPK1 with specific lentiviral shRNA in mouse PMN cultures (mPMNs). ***D***, Quantification of RIPK1 protein levels. RIPK1 protein is significantly increased in Tg SOD1^G93A^ samples compared to Tg SOD1^WT^ (*p* = 0.0279) and NTg (*p* = 0.0033) mice. Results are presented as mean ± SEM. Statistical analysis was performed via one-way ANOVA followed by Tukey’s *post hoc* analysis; *n* = 3 biological replicates per genotype. ***E***, Western blotting (RIPA) for RIPK3 showed no specific signal at the expected 55 kDa in spinal cord (NTg and Tg SOD1^G93A^). Non-specific band at 47 kDa is designated as an asterisk (*). NTg spleen: positive control tissue, *Ripk3^−/−^* spleen and Tg SOD1^G93A^;*Ripk3^−/−^*spinal cord: negative control tissue. ***F***, Western blotting (urea) for RIPK3 antibody showed no specific signal at the expected 55 kDa in spinal cord (NTg and Tg SOD1^G93A^). *Ripk3^−/−^* spinal cord: negative control tissue. ***G***, Western blotting (RIPA) for MLKL showed no specific signal at the expected 55 kDa in spinal cord. NIH 3T3: positive control cell lysate. ***H***, Western blotting (urea) for MLKL showed no specific signal at the expected 55 kDa in spinal cord (NTg and Tg SOD1^G93A^). NIH 3T3: positive control cell lysate. ***I***, Western blotting for p-MLKL (RIPA) showed no signal at the expected 55 kDa. TSZ-treated L929 cells: control cell lysate.

As a first step toward confirming necroptosis in ALS mice, we compared *Ripk1*, *Ripk3*, and *Mlkl* expression levels by qPCR in the lumbar spinal cord of Tg SOD1^G93A^ mice at two time points, ranging from early-symptomatic (i.e., 12 weeks of age) to near end-stage paralysis (i.e., 15 weeks of age; hereafter referred to as symptomatic), and in age-matched Tg SOD1^WT^ mice and NTg littermates. In early-symptomatic Tg SOD1^G93A^ mice, only *Ripk3* mRNA was significantly upregulated as compared to age-matched Tg SOD1^WT^ and NTg mice (Fig. 1*A*). In contrast, in symptomatic Tg SOD1^G93A^ mice, both *Ripk1* and *Ripk3* mRNA were significantly increased (Fig. 1*B*), whereas *Mlkl* mRNA levels remained unchanged.

To assess protein levels of these necroptosis factors, we first confirmed the specificity of two anti-RIPK1 antibodies in PMN cultures from WT E12.5 mouse embryos by silencing *Ripk1* (78% of control as determined by RT-qPCR). With the anti-RIPK1 antibody AB_394014, the band detected on the Western blot at the expected RIPK1 molecular mass of 74 kDa was reduced (Fig. 1*C*). Similar results were obtained for the second anti-RIPK1 antibody AB_397831 (data not shown). Using these validated antibodies, we then ran Western blots of mouse spinal cord samples and found a significant increase in RIPK1 protein levels in symptomatic, but not early-symptomatic (data not shown) Tg SOD1^G93A^ mice in comparison with Tg SOD1^WT^ and NTg mice, in tissue samples processed with RIPA (Fig. 1*C*,*D*) and urea (data not shown) extraction buffers.

For RIPK3 antibody validation, we used the spleen as a positive control given the high expression levels of RIPK3 in this organ in WT mice (Wang et al., 2016). Spleen tissue extracts from *NTg;Ripk3^+/+^* mice revealed two prominent bands, at ∼45kDa and at ∼55 kDa (Fig. 1*E*). In spleen tissue extracts from NTg;*Ripk3^−/−^* mice, the ∼55-kDa band, corresponding to the known molecular mass of RIPK3, was significantly reduced while the ∼45-kDa band (probably possibly a cleavage product) disappeared (Fig. 1*E*). We did not observe a similar ∼45-kDa band and detected only a faint and inconsistent ∼55-kDa band in spinal cord lysates from Tg SOD1^G93A^ and NTg mice with RIPA buffer only (Fig. 1*E*). Of note, non-specific bands at ∼47 kDa (with RIPA buffer; Fig. 1*E*) and ∼52 kDa (with urea buffer; Fig. 1*F*) were prominent and consistently observed in both NTg and Tg SOD1^G93A^ mice.

To validate the anti-MLKL antibody, we used extracts from mouse NIH 3T3 cells, which express high levels of MLKL protein and observed a 54-kDa band corresponding to molecular mass of the MLKL (Fig. 1*G*,*H*). However, using this same antibody we did not detect this band in spinal cord of NTg or Tg SOD1^G93A^ mice with either RIPA or urea buffer (Fig. 1*G*,*H*). With urea extraction, we detected a band at ∼52 kDa, which was below the validated ∼54-kDa band in the NIH 3T3 lysate (Fig. 1*H*). To generate a positive control for p-MLKL, we induced necroptosis with TNFα/zVAD/SMAC in L929 cells as done by Hitomi et al. (2008). While the extracts from the necroptosis-induced L929 cells showed the expected ∼54-kDa band using our anti-p-MLKL antibody, we did not detect any such band in spinal cords of NTg or Tg SOD1^G93A^ mice (Fig. 1*I*). These results led us to conclude that levels of RIPK3, MLKL and p-MLKL in spinal cord of NTg or Tg SOD1^G93A^ mice are below the detection limits of the validated antibodies in Western blotting. Thus, RIPK1 was the only necroptosis pathway protein unambiguously detected and upregulated in Tg SOD1^G93A^ mice. The discrepancy between levels of *Ripk3* transcripts and protein needs further elucidation.

### RIPK1 expression in human ALS brain samples

We next asked whether a similar RIPK1 upregulation is observed in postmortem tissue from ALS patients. For this purpose, we used brain homogenates from Brodmann’s area 4, the primary motor cortex, from patients with sporadic ALS (*n* = 6), SOD1 ALS (*n* = 2), and age-matched control patients (*n* = 4). Patient information can be found in Table 1.

**Table 1.** ALS patient samples history

Disease	Sex	Age at death	PMI cold (HH:MM)	PMI frozen (HH:MM)	Age at onset	Disease duration (months)	Notes
NON-ALS #1	M	82	4:55	11:20	.	.	Neuro normal
NON-ALS #2	F	54	6:41	16:36	.	.	Neuro normal
NON-ALS #3	F	62	4:33	4:12	.	.	Hypoxic ischemic encephalopathy
NON-ALS #4	M	60	N/A	10:08	.	.	Neuro normal
SALS #5	M	67	4:40	19:20	65	19	Right lower extremity onset
SALS #6	M	56	4:53	10:57	54	18	Lower extremity onset
SALS #7	F	72	3:01	19:57	70	29	Diffuse weakness, proximal involvement
SALS #8	M	56	N/A	13:17	54	33	Lower extremity onset, used high dose steroids (prednisone, decadron, testosterone), angiogenin mutation (I70V)
SALS #9	F	71	N/A	5:11	70	30	Bulbar onset
SALS #10	F	69	5:30	5:00	67	18	Bulbar onset
FALS #11	F	52	12:45	4:00	50	24	SOD1 mutation (L144F)
FALS #12	F	32	N/A	5:10	29	30	SOD1 mutation (A5V)

SALS, sporadic ALS; FALS; familial ALS; M, male; F, female, N/A, not available; PMI, postmortem interval.

**Figure 2.**
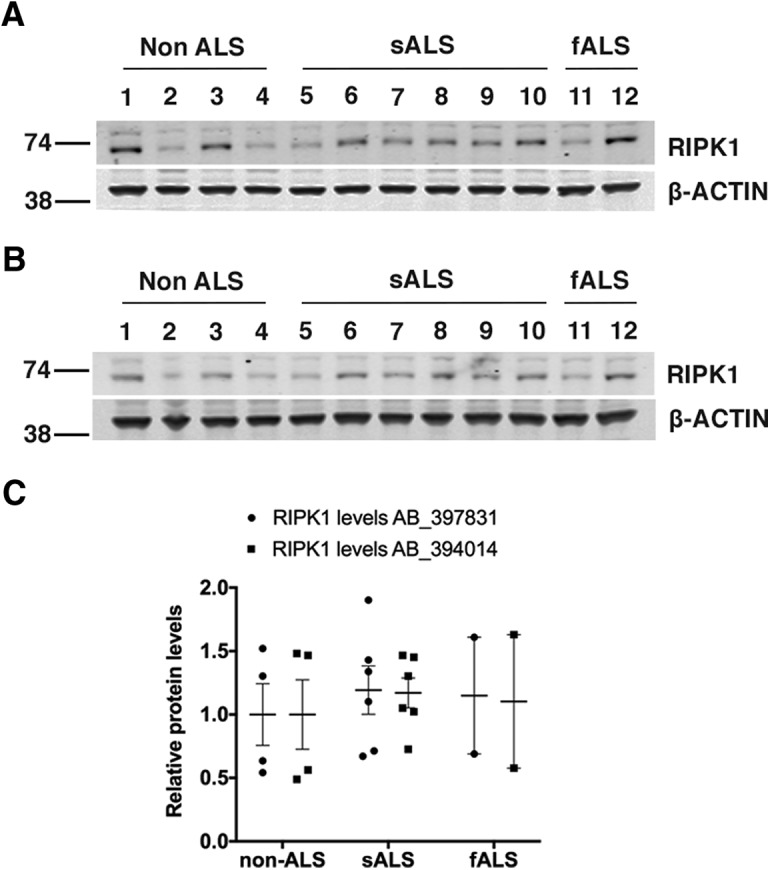
RIPK1 expression in brain cortex from ALS patients. Postmortem motor cortex (Brodmann’s area 4) from sporadic ALS, SOD1 ALS, and non-ALS human brains was homogenized and processed for RIPK1 protein expression. Western blotting for RIPK1 protein. Two different antibodies against RIPK1 were used (***A***, AB_397831; ***B***, AB_394014). ***C***, Quantification of RIPK1 protein levels. β-ACTIN, loading control. No significant differences were detected for RIPK1 between sporadic ALS, SOD1 ALS, and non-ALS human brain samples. Results are presented in a scatter dot plot. Line = mean. Statistical analysis was performed via Student’s *t* test: *t*_(10)_ = 0.579, *p* = 0.575; *n* = 4; non-ALS, *n* = 6; sporadic ALS, and *n* = 2; SOD1 ALS.

Using two different validated antibodies against RIPK1, we detected the expected ∼74-kDa band. However, both antibodies showed that RIPK1 expression was faint, highly variable across all samples (Fig. 2) and not significantly different between ALS and controls. In addition, we did not observe any significant correlations (linear regression, R ≤ 0.05, *p* ≥ 0.75) between age at onset, duration of the disease, postmortem delay and RIPK1 expression levels on Western blotting (data not shown).

### Ripk3 mediates MN death in an *in vitro* model of ALS

**Figure 3.**
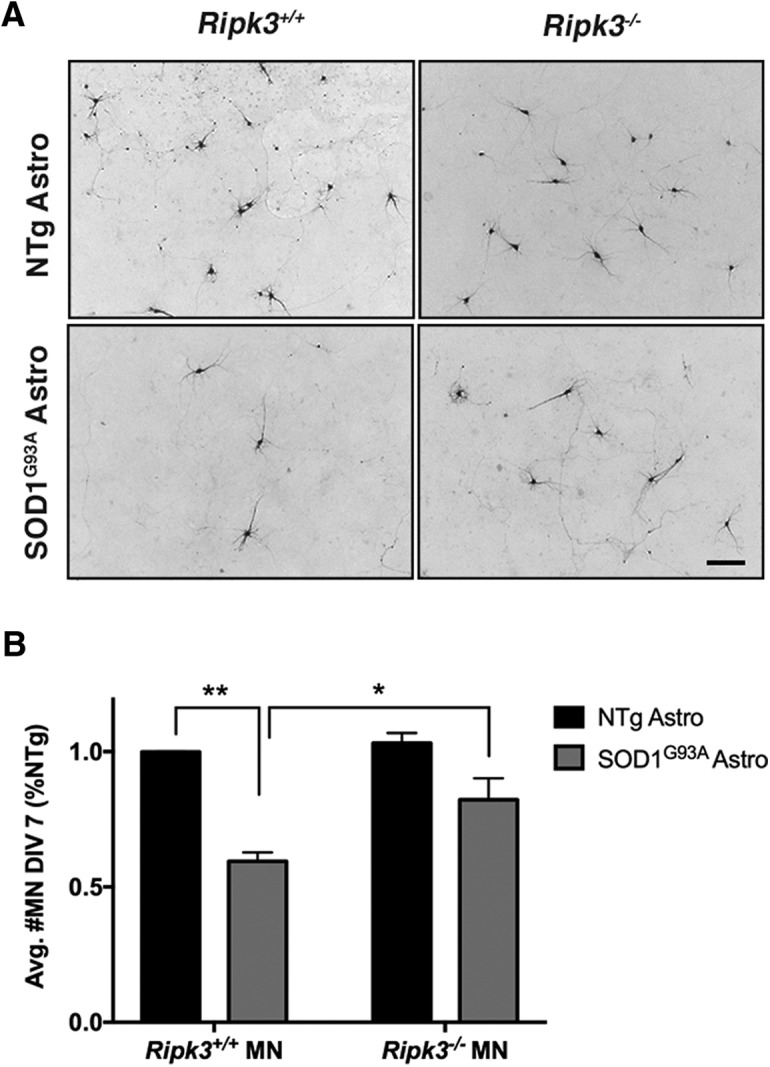
*Ripk3^−/−^*MNs are resistant to Tg SOD1^G93A^ astrocyte-mediated toxicity. MNs, isolated from E12.5 *Ripk3^+/+^*or *Ripk3^−/−^*mice, were co-cultured on primary astrocyte monolayers from Tg SOD1^G93A^ or NTg mice for 7 d. ***A***, Representative images of MNs assessed using SMI32 immunolabeling. Scale bar: 50 μm. ***B***, Quantification of MN number. *Ripk3^+/+^*MN number was significantly reduced on SOD1^G93A^ astrocytes (*p* = 0.0013) compared to NTg. *Ripk3^−/−^*MN number did not differ between NTg or SOD1^G93A^ astrocytes and was significantly increased (*p* = 0.0370) compared to *Ripk3^+/+^* MN number on SOD1^G93A^ astrocytes. Results are presented as a mean ± SEM. Statistical analysis was performed via two-way ANOVA; *n* = 3 biological replicates per genotype. *p ≤ 0.05; **p ≤ 0.01.

Previous studies have shown that RIPK1 contributes to MN death in *in vitro* models of ALS (Re et al., 2014). To expand characterization of the machinery of necroptosis in *in vitro* models, we determined the involvement of RIPK3 using constitutive knock-out mice (*Ripk3^-/^* ^-^) deficient in this kinase (Newton et al., 2004), which develop normally to adulthood without any observed defects in weight gain or fertility, and no histologic defects in major organs, including the CNS (Newton et al., 2004). We first assessed mRNA expression of *Ripk3* in the brain and spinal cord from *Ripk3^−/−^* mice and their WT counterparts. As expected, no expression of *Ripk3* mRNA transcript was detected in *Ripk3^−/−^* CNS (data not shown). To assess the role of Ripk3 in astrocyte-mediated MN toxicity in the SOD1^G93A^ model, we co-cultured MNs obtained from *Ripk3* ^−/−^ and *Ripk3^+/+^* mice with astrocytes from Tg SOD1^G93A^ and NTg mice (Nagai et al., 2007; Re et al., 2014; Ikiz et al., 2015) and monitored the loss of MN over time. We found that MNs deficient in *Ripk3* survived significantly longer than WT MNs co-cultured with Tg SOD1^G93A^ astrocytes (Fig. 3). These results indicate that the constitutive deletion of *Ripk3* protects embryonic MNs against the deleterious effects of SOD1^G93A^ astrocytes, similar to what has been shown for *RIPK1* (Re et al., 2014). Therefore, both RIPK1 and RIPK3 appear to be significant contributors for MN death in this *in vitro* model of ALS. Of note, here, we did not test the potential effect of Ripk3 deletion in SOD1^G93A^ astrocytes on MN death, since our previous work (Re et al., 2014) established that RIPK1 contributes to motor neurodegeneration via a cell autonomous mechanism.

### Genetic deletion of *Ripk3* does not alter the pathologic hallmarks of Tg SOD1^G93A^ mice

Guided by these *in vitro* data, we expanded our investigation of necroptosis in ALS using an *in vivo* model of the disease. We posited that if the necroptosis contributes to neurodegeneration in Tg SOD1^G93A^ mice, then inhibition of this pathway should mitigate the ALS-like phenotype in these animals. To achieve this, we generated *Ripk3* homozygous knock-out mice in the Tg SOD1^G93A^ background. We chose to use the Tg SOD1^G93A^;*Ripk3^−/−^*mouse as a model for several reasons: first, *Ripk3^−/−^* mice have been shown to be resistant to a variety of necroptosis stimuli (Vanlangenakker et al., 2012; Geserick et al., 2015; Zhao et al., 2017); second, mutant *Ripk3^−/−^*mice are healthy, while mutant *Ripk1^−/−^ mice* die soon after birth; and lastly, RIPK3 is increasingly recognized as more necroptosis-specific than RIPK1, which also triggers inflammatory responses through activation of the NFkB pathway (Festjens et al., 2007; Ofengeim and Yuan, 2013).

**Figure 4.**
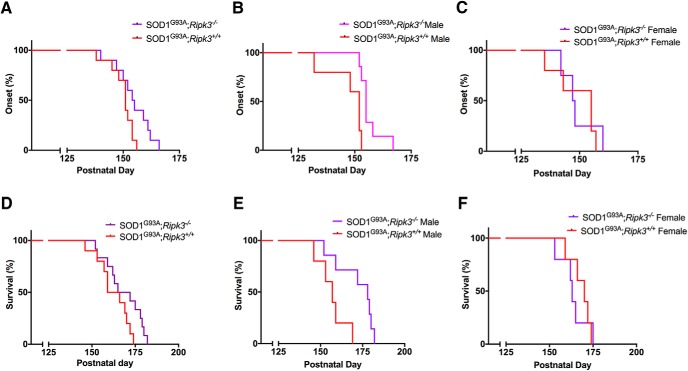
Onset and survival in Tg SOD1^G93A^*;Ripk3^−/−^* mice. Mice were separated by genotype and sex for onset (%) and survival (%), and data were plotted as Kaplan–Meier curves. ***A***, Onset in Tg SOD1^G93A^*;Ripk3^−/−^* (median = 155) and Tg SOD1^G93A^*;Ripk3^+/+^* (median = 151), *p* = 0.06; male and female mice. ***B***, Onset in Tg SOD1^G93A^*;Ripk3^−/−^* (median = 155) and Tg SOD1^G93A^*;Ripk3^+/+^* (median = 152), *p* = 0.005; male mice. ***C***, Onset in Tg SOD1^G93A^*;Ripk3^−/−^* (median = 148) and Tg SOD1^G93A^*;Ripk3^+/+^* (median = 155), *p* = 0.836; female mice. ***D***, Survival in Tg SOD1^G93A^*;Ripk3^−/−^* (median = 169) and Tg SOD1^G93A^*;Ripk3^+/+^* (median = 163), *p* = 0.145; male and female mice. ***E***, Survival in Tg SOD1^G93A^*;Ripk3^−/−^* (median = 178) and Tg SOD1^G93A^*;Ripk3^+/+^* (median = 157), *p* = 0.017; male mice. ***F***, Survival in Tg SOD1^G93A^*;Ripk3^−/−^* (median = 163) and Tg SOD1^G93A^*;Ripk3^+/+^* (median = 170), *p* = 0.340; female mice. For ***A***, ***D***, 10 Tg SOD1^G93A^*;Ripk3^+/+^* mice and 12 Tg SOD1^G93A^*;Ripk3^−/−^* mice. For ***B***, ***E***, 5 Tg SOD1^G93A^*;Ripk3^+/+^* mice and 7 Tg SOD1^G93A^*;Ripk3^−/−^* mice. For ***C***, ***F***, 5 Tg SOD1^G93A^*;Ripk3^+/+^* mice and 5 Tg SOD1^G93A^*;Ripk3^−/−^* mice. Statistical analysis was performed via log-rank Mantel–Cox test.

We first confirmed that *Ripk3^−/−^* mice survived, bred and gained weight like their WT counterparts. In Tg SOD1^G93A^ mice, onset of disease is defined as the age at which a mouse loses 10% of its peak weight (Liu et al., 2005). Using this parameter, we initially found no significant difference in the age of onset between Tg SOD1^G93A^;*Ripk3^−/−^*and Tg SOD1^G93A^;*Ripk3^+/+^* mice (Fig. 4*A*). However, when the data were stratified according to sex, male Tg SOD1^G93A^;*Ripk3^−/−^* mice, but not female Tg SOD1^G93A^;*Ripk3^−/−^*mice, exhibited a delayed onset compared to their Tg SOD1^G93A^;*Ripk3^+/+^*counterparts (Fig. 4*B*,*C*). The same effect of Ripk3 elimination on onset was observed for survival, defined as the time between birth to the loss of righting ability (Gurney et al., 1994). There was no significant difference in survival overall between Tg SOD1^G93A^;*Ripk3^−/−^*and Tg SOD1^G93A^;*Ripk3^+/+^* mice (Fig. 4*D*), however male Tg SOD1^G93A^;*Ripk3^−/−^* mice survived significantly longer than male Tg SOD1^G93A^;*Ripk3^+/+^*controls (Fig. 4*E*). Again, female Tg SOD1^G93A^;*Ripk3^−/−^*and Tg SOD1^G93A^;*Ripk3^+/+^* mouse survival did not differ (Fig. 4*F*).

**Figure 5.**
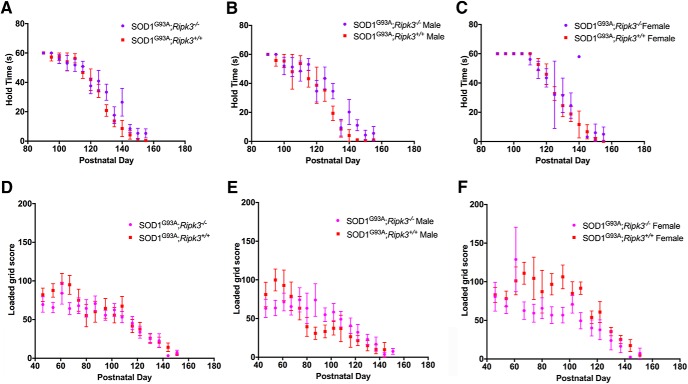
Functional motor tests on Tg SOD1^G93A^*;Ripk3^−/−^* mice. Mice were separated by genotype and sex for grip strength analysis. ***A***, Inverted grid score in Tg SOD1^G93A^*;Ripk3^−/−^* and Tg SOD1^G93A^*;Ripk3^+/+^* male and female mice. ***B***, Inverted grid score in Tg SOD1^G93A^*;Ripk3^−/−^* and Tg SOD1^G93A^*;Ripk3^+/+^* male mice. ***C***, Inverted grid score in Tg *SOD1^G93A^;Ripk3^−/−^* and Tg *SOD1^G93A^;Ripk3^+/+^* female mice. ***D***, Loaded grid score in Tg SOD1^G93A^*;Ripk3^−/−^* and Tg SOD1^G93A^*;Ripk3^+/+^* male and female. ***E***, Loaded grid score in Tg SOD1^G93A^*;Ripk3^−/−^* and Tg SOD1^G93A^;*Ripk3^+/+^* male mice. ***F***, Loaded grid score in Tg SOD1^G93A^*;Ripk3^−/−^* and Tg SOD1^G93A^*;Ripk3^+/+^* female mice. For ***A***, ***D***, 14 Tg SOD1^G93A^*;Ripk3^+/+^* mice and 17 Tg SOD1^G93A^*;Ripk3^−/−^* mice. For ***B***, ***E***, 8 Tg SOD1^G93A^*;Ripk3^+/+^* mice and 10 Tg SOD1^G93A^*;Ripk3^−/−^* mice. For ***C***, ***F***, 6 Tg SOD1^G93A^*;Ripk3^+/+^* mice and 7 Tg SOD1^G93A^*;Ripk3^−/−^* mice. Statistical analysis was performed by unpaired *t test*, two-tailed in selected time points using *post hoc* Bonferroni correction.

Motor performance was assessed from presymptomatic to end-stage by inverted and loaded grids (Barnéoud et al., 1997; Oliván et al., 2015). For both tests, no differences between Tg SOD1^G93A^;*Ripk3^-^* ^/-^ and Tg SOD1^G93A^;*Ripk3^+/+^* mice were detected (Fig. 5*A*,*D*), and here, there was also no difference between the two genotypes when the data were stratified by sex (Fig. 5*B*,*C*,*E*,*F*). We recognize that the high variability of the two tests and the size of our mouse cohorts lead to a high risk of false negative findings. Yet, given the small effect size at the time points at which Tg SOD1^G93A^;Ripk3^−/−^ and Tg SOD1^G93A^;Ripk3^+/+^ mice seem to diverge, demonstrating statistical significance of the differences between these genotypes by increasing the number of mice per group would be of dubious value.

**Figure 6.**
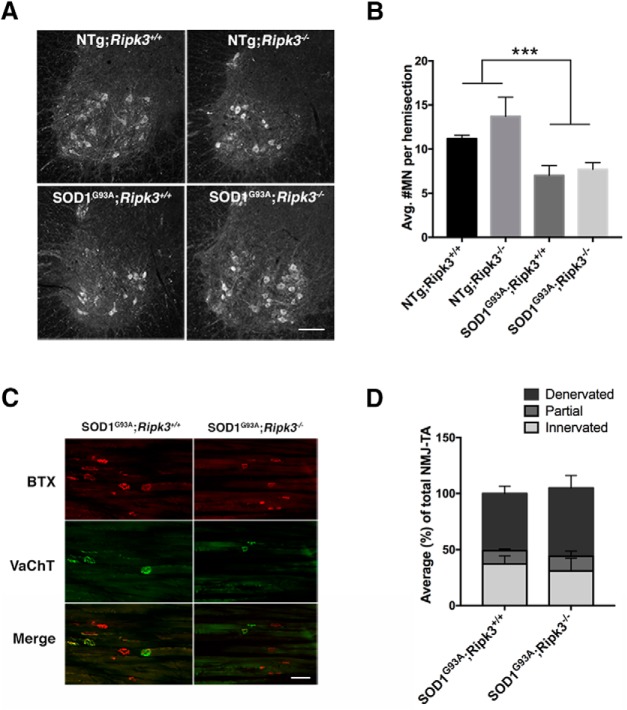
MN number and NMJ innervation is not improved in Tg SOD1^G93A^*;Ripk3^−/−^* mice. ***A***, Representative images of ChAT+ cells in lumbar (L4-L5) ventral horns from P140 (close to end-stage) mice (fluorescent microscope; 10× magnification). Scale bar: 50 μm. ***B***, Quantification of MN number, expressed as average number of neurons per hemisection (20 μm). Statistical significant difference was detected between NTg (*Ripk3^+/+^* and *Ripk3* ^−/−^) and Tg (SOD1^G93A^*;Ripk3^+/+^* and SOD1^G93A^*;Ripk3^−/−^*), *p* < 0.0001. No difference was detected between Tg SOD1^G93A^*;Ripk3^−/−^* and Tg SOD1^G93A^;*Ripk3^+/+^* mice. Results are presented as a mean ± SEM. Statistical analysis was performed via two-way ANOVA followed by Newman–Keuls *post hoc* test; *n* = 3 biological replicates per genotype. ***C***, Representative images of NMJ assessed by the expression of BTX (red, postsynaptic) and VAChT (green, presynaptic) in the TA muscle of P120 mice. Left column, Tg SOD1^G93A^*;Ripk3^+/+^*. Right column, Tg SOD1^G93A^*;Ripk3^−/−^* mice. Scale bar: 100 μm. ***D***, Quantification of NMJs from 20-μm sections (*n* = ∼100 NMJs). NMJs were categorized as innervated (complete colocalization of BTX and VAChT), partial (partial colocalization of BTX and VAChT), or denervated (no colocalization between BTX and VAChT) and are presented as a percentage of the total NMJ number counted. No difference was observed in the number of innervated, partially innervated, and denervated NMJs between Tg SOD1^G93A^*;Ripk3^−/−^*and Tg SOD1^G93A^*;Ripk3^+/+^* mice. Results are presented as a mean ± SEM. Statistical analysis was performed via unpaired Student’s *t* test, two-tailed; *n* = 3 biological replicates per genotype. ***p ≤ 0.001.

Next, we assessed the morphological hallmarks of the disease in Tg SOD1^G93A^;*Ripk3^−/−^*mice, limiting our analysis to the male mutants in which an improvement in onset and survival was observed. Typically, Tg SOD1^G93A^ mice exhibit ∼50% MN loss in the lumbar spinal cord by end-stage (Higgins et al., 2002), preceded by extensive NMJ denervation (Fischer et al., 2004), especially in the TA muscle (Pun et al., 2006). The number of MNs in lumbar segments 4 and 5 of the mouse spinal cord, including those that innervate the TA muscle, was quantified at P140 (Fig. 6*A*), a timepoint where significant MN loss can be observed in the B6.Cg-Tg(SOD1*G93A)1Gur/J mice. A two-way ANOVA showed no significant difference in the number of MNs at P140 Tg SOD1^G93A^;*Ripk3^−/−^*compared to Tg SOD1^G93A^;*Ripk3^+/+^*male mice, although both groups had significantly fewer MNs than their NTg counterparts (Fig. 6*B*). In addition, no difference in MN number was found at P140 between NTg;*Ripk3^−/−^*and NTg;*Ripk3^+/+^*male mice (Fig. 6*B*). For the status of NMJ innervation in the TA muscles of Tg SOD1^G93A^;*Ripk3^−/−^*and Tg SOD1^G93A^;*Ripk3^+/+^*male mice, an earlier time point (P120) was chosen, since NMJ denervation precedes MN cell death in Tg SOD1^G93A^ mice. Similar to MN counts, no difference between the number of innervated, denervated and partially-innervated NMJs (see *Methods* for definitions) was found when comparing Tg SOD1^G93A^;*Ripk3^−/−^* to Tg SOD1^G93A^;*Ripk3^+/+^*male mice (Fig. 6*C*,*D*). Therefore, the observed delay in onset and extension in survival of male Tg SOD1^G93A^;*Ripk3^−/−^*compared to male Tg SOD1^G93A^;*Ripk3^+/+^* mice was not associated with improvements in either motor performance or neuropathological hallmarks of the disease.

**Figure 7.**
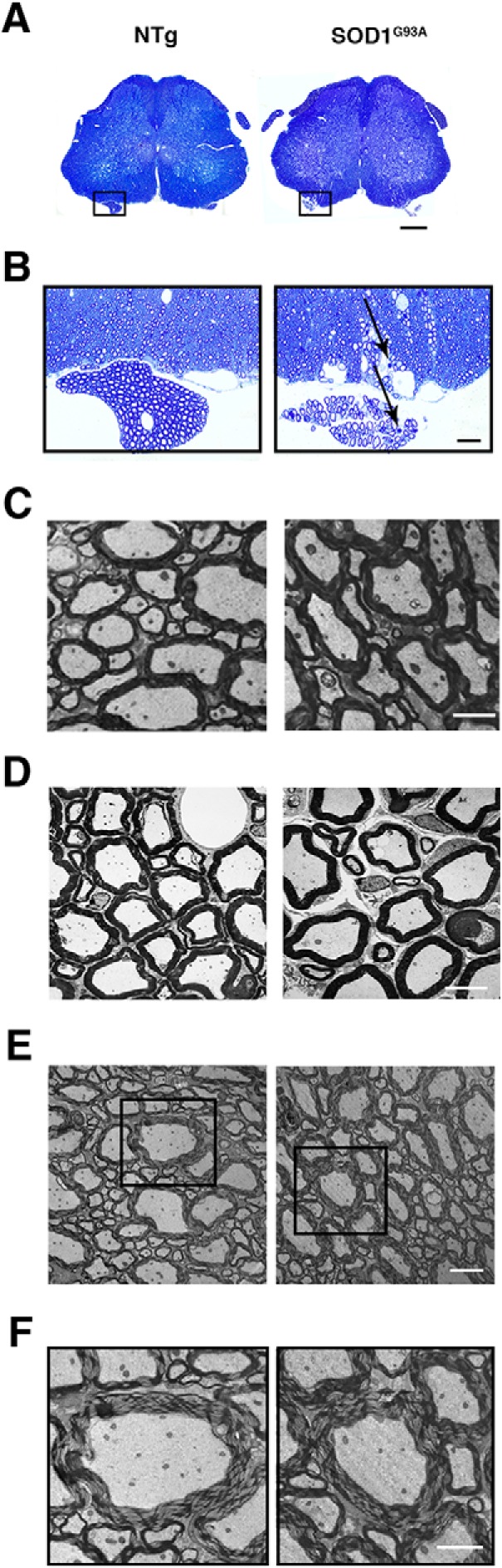
MN myelination morphology is not impaired in Tg SOD1^G93A^ P90 mice. ***A***, Gross morphology of the spinal cords (L1-L5) in transverse sections. ***B***, Enlarged boxed areas in ***A*** indicate areas of white matter of ventral horn with adjacent ventral root. Note myelin degeneration only in Tg SOD1^G93A^ (arrows in ***B***, marked only few). ***A***, ***B***, Semithin sections, stained with toluidine blue. ***C***, Ultrastructure of myelinated fibers in white matter of ventral horn. ***D***, Ultrastructure of ventral roots. Note that myelin morphology is not impaired in both genotypes in ***C***, ***D***. All images in the horizontal panels were obtained at the same magnification. ***E***, Ultrastructure of white matter ventral horn. ***F***, Enlarged boxed areas in ***E***. Note the decompacted morphology of myelin in both genotypes. Vertical columns correspond to the mice genotype indicated at the top. Scale bars: 0.5 cm (***A***), 25 μm (***B***), 10 μm (***C–E***), 5 μm (***F***).

Since no differences were detected in MN survival and muscle innervation between the Tg SOD1^G93A^;*Ripk3^+/+^*and Tg SOD1^G93A^;*Ripk3^−/−^* mice, we compared the motor axons myelin compaction. This idea was prompted by the report of Ito et al. (2016) that myelin decompaction may serve as a proxy of necroptosis in ALS. Therefore, as a first step, we used electron microscopy to examine lumbar spinal cord and ventral roots from NTg and early-symptomatic Tg SOD1^G93A^ mice (Fig. 7). As expected and shown by others (Guo et al., 2010; Vinsant et al., 2013), early-symptomatic Tg SOD1^G93A^ mice showed ultrastructural changes in the ventral horn, funiculus, and roots including both shrunken and swollen MNs, large vacuoles and degenerating axons (Fig. 7*B*). In the case of spared myelinated fibers, the myelin sheath was preserved in the anterior funiculus and ventral roots in Tg SOD1^G93A^ mice (Fig. 7*C*,*D*). However, myelin preservation varied within as well as between mice, and myelin alterations reminiscent of myelin decompaction were observed in both Tg SOD1^G93A^ and NTg mice (Fig. 7*E*,*F*).

**Figure 8.**
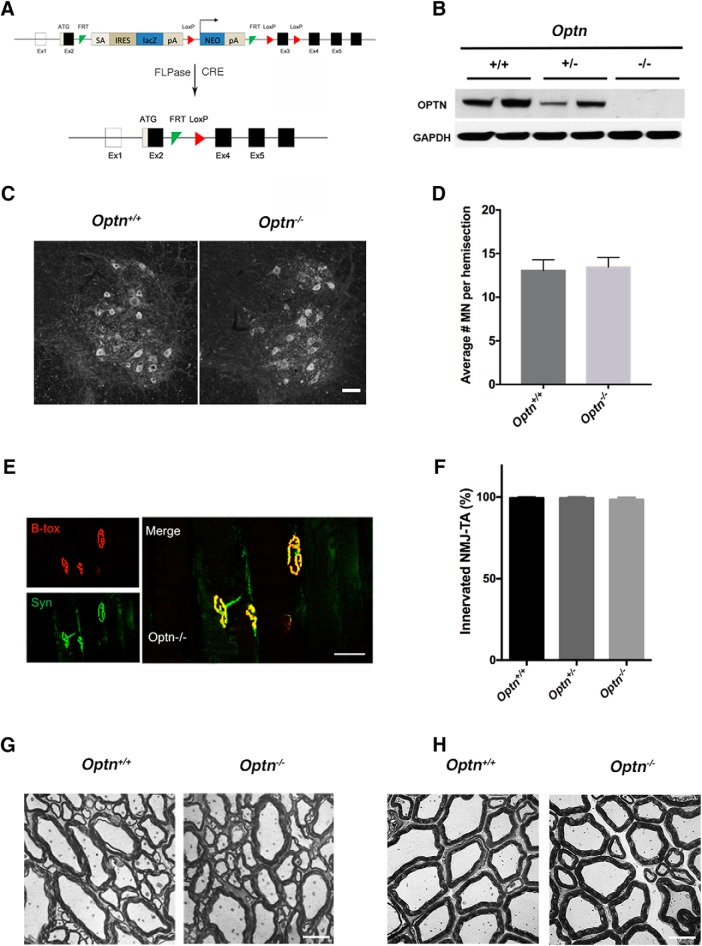
Characterization of the *Optn^−/−^* mouse. ***A***, Schematic of the *Optn^−/−^* mice. *Optn* knock-out allele was generated by two steps crossing of FLPase and CRE mice separately with *Optn* ^tm1a(EUCOMM)Wtsi^ mice to trim out the critical exon. ***B***, Western blotting for OPTN expression in *Optn^+/−^* (+/−), *Optn^−/−^* (−/−), and control (+/+) spinal cord tissue. GAPDH, loading control. ***C***, Representative images of ChAT+ cells in lumbar ventral spinal cord (L4-L5) from one-year-old *Optn^+/+^* and *Optn^−/−^* mice. Scale bar: 50 μm. ***D***, Quantification of medial motor column (MMC) MN number, expressed in average number of neurons per hemisection. Results are presented as a mean ± SEM. Statistical analysis was performed via unpaired Student’s *t* test; *n* = 3 biological replicates per genotype. ***E***, Representative images of NMJ, of the TA muscle, assessed by the expression of BTX (red, postsynaptic) and Syn (green, presynaptic) from one-year-old *Optn^−/−^* mice. Colocalization of BTX and Syn represents an innervated NMJ. Scale bar: 50 μm. ***F***, Quantification of innervated NMJs (%) in the *Optn^+/+^*, *Optn^+/−^*, and *Optn^−/−^* mice. Results are presented as mean ± SEM. Statistical analysis was performed via unpaired Student’s *t* test, two-tailed; *n* = 3 biological replicates per genotype. ***G***, Ultrastructure of white matter ventral horn in the *Optn^+/+^* and *Optn^−/−^* mice. ***H***, Ultrastructure of ventral roots in the *Optn^+/+^* and *Optn^−/−^* mice. Note that myelin does not differ in both genotypes of mice in ***G***, ***H***. Scale bar: 10 μm (***G***, ***H***).

In addition, since the link between necroptosis and myelin decompaction was reported to be more pronounced in mutant *Optn^−/−^*than in Tg SOD1^G93A^ mice (Ito et al., 2016), we generated a mouse with homozygous germline deletion of the *Optn* gene (Fig. 8*A*). After verifying the complete knock-out of the optineurin protein in *Optn^−/−^*mice (Fig. 8*B*), we then monitored *Optn^−/−^* mice development and found no significant difference compared to their WT littermates in terms of newborn birth weight, appearance and behavior (data not shown). Furthermore, up to two years of age, we recorded no difference in survival and behavior between *Optn^−/−^*and *Optn^+/+^*mice (data not shown). Similar to Ito et al. (2016), we observed no difference in spinal MN number between one-year old *Optn^−/−^*mice and their aged-matched WT littermates (Fig. 8*B*,*C*). In contrast to Ito et al. (2016), we did not observe any denervation of the TA muscle and observed similar density of NMJs in one-year old *Optn^−/−^*mice as compared to their aged-matched WT littermates (Fig. 8*D*,*E*). We were unable to detect any genotype-specific alterations in myelin compaction in the ventral white matter (Fig. 8*G*) or in the ventral roots (Fig. 8*H*). Therefore, while OPTN loss of function has been a proposed mechanism by which mutations in *OPTN* may lead to ALS (Maruyama et al., 2010), our data argue that the constitutive loss of OPTN at the germline level is not sufficient for the generation of an ALS-like phenotype in mice.

## Discussion

Necroptosis has been implicated in the pathogenesis of ALS. Here, to confirm the role of this form of PCD in MN disease, we assessed the expression of its three known core factors in two distinct animal models of ALS, and in ALS patient tissue. Transcripts for *Ripk1*, *Ripk3*, and *Mlkl* were all detected in mouse spinal cord of NTg, Tg SOD1^WT^ and Tg SOD1^G93A^ mice. However, only mRNA for *Ripk1* and *Ripk3* (but not for *Mlkl*) were increased in the spinal cord of symptomatic Tg SOD1^G93A^ mice compared to control animals. In contrast to the levels of the mRNAs, only RIPK1 protein increased in the spinal cord of symptomatic Tg SOD1^G93A^ compared to age-matched Tg SOD1^WT^ and NTg mice. In our hands, RIPK3, MLKL and p-MLKL were either faintly or not at all detectable by Western blot analysis of spinal cord samples from Tg SOD1^G93A^ and NTg mice, even after loading large amounts of protein or using high stringency buffers. Our findings are in line with those of Kanno et al. (2015) who also failed to detect RIPK3 in the spinal cord of adult WT mice by immunolabeling, and consistent with the work of Wu et al. (2013), which likewise did not find detectable levels of MLKL in various mouse tissues including the brain. Together, these negative results are in striking contrast to those of Ito et al. (2016) where the same methods and immunoreagents were used to consistently detect robust signals for RIPK3, MLKL and p-MLKL in the spinal cord of both NTg and early-symptomatic Tg SOD1^G93A^ mice.

Notably, the levels of RIPK1 in human postmortem samples were highly variable, with no clear trend among patients compared to controls. This variability in RIPK1 content may be related to varying degrees of neuropathological changes in the study samples and the severity and length of the agonal hypoxia in each case. Indeed, hypoxia can induce a strong upregulation of RIPK1 (Degterev et al., 2005; Vieira et al., 2014) making the detection of any disease-specific effect challenging. Furthermore, it was recently found that transcriptional upregulation can occur postmortem, especially in the brain (Pozhitkov et al., 2017), and that many of the transcripts upregulated during the first postmortem hour encode for proteins involved in cell death and inflammation. These findings urge caution in our interpretation of studies of cell death factors in postmortem tissues, including those that mediate necroptosis.

Since only a small number of MNs are degenerating at any given time in ALS patients or in mouse models of the disease, the molecular expression of MN-derived necroptosis in whole tissue extracts may be challenging to detect by Western blotting. Moreover, since the engagement of necroptosis requires the phosphorylation of RIPK1, RIPK3, and MLKL, these proteins’ post-translational modifications and not their abundance per se is critical. In our hands, the only available antibody raised against an active form of a necroptosis determinant was an anti-phospho-MLKL antibody, which failed to generate any specific signal in mouse tissues, hence precluding us from formulating trustworthy conclusion about whether or not necroptosis is activated in spinal cords of Tg SOD1^G93A^ mice during MN degeneration. Furthermore, also in contrast to two other studies (Ito et al., 2016; Caccamo et al., 2017), we failed to find that the RIPK1 and RIPK3 antibodies, validated for Western blotting, generated specific signal in immunohistochemistry either in mouse CNS sections or cultured MNs (data not shown).

In light of the above, we decided to target a specific determinant of necroptosis and assess the effect of its elimination on the ALS phenotype in Tg SOD1^G93A^ mice to determine more definitely if necroptosis is involved in the motor neurodegenerative process. We chose to target RIPK3 since, in contrast to RIPK1, it has no known function outside of necroptosis (Festjens et al., 2007; Ofengeim and Yuan, 2013; Newton, 2015). To reproduce the experimental conditions used in Ito et al. (2016), we took a genetic approach using constitutive RIPK3 knock-out mice (Newton et al., 2004). Like RIPK1 inhibition, we found that the constitutive loss of RIPK3 in cultured mouse MNs protected against the deleterious effects of SOD1^G93A^-expressing astrocytes *in vitro*. However, the elimination of RIPK3 had no effect on the ALS-like phenotype in Tg SOD1^G93A^ mice as measured by onset of disease, survival, motor performance, lumbar MN number, and TA NMJ denervation. Of note, improvements in onset and survival were observed in male Tg SOD1^G93A^;Ripk3^−/−^ mice as compared to male Tg SOD1^G93A^;Ripk3^+/+^ mice. These sex-differences did not correlate with any of the studied neuropathological parameters, nevertheless the benefit of RIPK3 elimination in male mutants may be significant and worthy of further investigations. This finding also illustrates the critical importance of stratifying experimental cohorts by sex. As for the lack of effect of RIPK3 deletion on the ALS-like motor phenotype, at least in female mice and on neuropathology in both sexes, we cannot exclude that the constitutive ablation of *RIPK3* was mitigated by unknown molecular compensation that was stronger in female and sufficient to mask signs of neurodegeneration in both sexes. For instance, particularly in females, in the absence of RIPK3, the MN death signal could proceed via alternate pathways, perhaps involving cooperation between RIPK1 and caspase-8 (Feltham et al., 2017) and this compensation may be more effective in female mice. Future studies in which RIPK3 deletion is combined with caspase-8 inhibition would be informative in this respect.

Although we did not see a significant effect of targeting necroptosis via RIPK3 on the ALS-like neuropathology, we nevertheless sought to examine the state of compaction of motor axon myelin, whose alteration was used by Ito et al. (2016) as a proxy for necroptosis in ALS mouse models. In contrast to that study, we only detect signs of myelin decompaction inconsistently in the ventral spinal white matter and ventral roots in both Tg SOD1^G93A^ and mutant *Optn^−/−^* mice, and more importantly, we observe this abnormal myelin morphology equally in NTg, Tg SOD1^G93A^, and mutant *Optn^−/−^* mice. Given that such an abnormal myelin appearance is a known artifact of myelin fixation (Condie et al., 1961; Feirabend et al., 1994; Möbius et al., 2016), we believe that the reported myelin sheath decompaction is the result of technical variability rather than a reflection of the disease process.

Collectively, our *in vivo* data call for further investigations targeting key determinants of necroptosis in models of ALS. To minimize the risk of compensatory mechanisms, the elimination of these factors should be done conditionally, after the postnatal period, and not constitutively as done here and in Ito et al. (2016). It would also be critical to obtain sensitive and specific immunoreagents to detect the active form of the drivers of necroptosis, especially at a cellular level. Lastly, stable and brain permeant small molecules can now be used to inhibit RIPK3 and other determinants of necroptosis pharmacologically. It would thus be quite important to test such compounds in animal models of ALS to determine whether necroptosis plays a pathogenic role in ALS. This is what Ito et al. (2016) have done with the kinase inhibitor of RIPK1, Nec1s, but since that study only reports on onset of motor dysfunction and on myelin decompaction, we believe that the potential therapeutic role of targetting key determinants of necroptosis in ALS and related neurodegenerative disorders remains to be established.

## Synthesis

Reviewing Editor: Robert Kalb, Northwestern University Decisions are customarily a result of the Reviewing Editor and the peer reviewers coming together and discussing their recommendations until a consensus is reached. When revisions are invited, a fact-based synthesis statement explaining their decision and outlining what is needed to prepare a revision will be listed below. The following reviewer(s) agreed to reveal their identity: Eric Huang.

Both reviewers were impressed by the work and the contribution it makes to the field. A

variety of additional analyses are recommended. If the Przedborski Lab has the requested data, it would be great to include them in the ms. If not, a reasonable verbal argument would suffice.

Reviews #1 comments

This study investigates the role of Ripk3 in neurodegeneration in the mouse SOD1-G93A ALS model. The authors showed that Ripk1 and Ripk3 mRNAs were elevated in the spinal cord of pre-symptomatic and symptomatic SOD1-G93A mice. To investigate the role of Ripk3 in neurodegeneration, the authors showed that Ripk3-/- motor neurons are more resistant to cell death induced by SOD1-G93A astrocytes. Interestingly, SOD1-G93A;Ripk3-/- male mice showed a modest delay in disease onset and better survival, whereas female SOD1-G93A;Ripk3-/- showed no difference compared to SOD1-G93A Tg mice.

Overall, this is an interesting study that directly addresses that role of Ripk3 in neurodegeneration in SOD1-G93A ALS model using both in vitro and in vivo approaches. The results are quite intriguing and show distinct differences compared to those from Ripk1-focused studies. However, there are a few areas that require further work to eliminate confusions and improve clarity in the presentation.

Major Comments:

1. Results in Figure 3 support the idea that, when co-cultured with SOD1-G93A astrocytes, Ripk3-/- motor neurons are more resistant to glial toxicity. However, the statistical analyses are incomplete. It will be important to make direct comparison between Ripk2+/+ and Ripk2-/- neurons when co-cultured with SOD1-G93A astrocytes and with WT astrocytes (for Ripk3-/- neurons). Given the potential role of Ripk3 in astrocytes, it will be important to determine whether SOD1-G93A;Ripk3-/- astrocytes are less toxicity to both wild type and Ripk3-/- motor neurons. Finally, in light of the gender difference observed in Figure 4, the authors should determine whether gender-specific differences can also be detected in Ripk3-/- motor neurons and astrocytes.

2. The potential gender difference in SOD1-G93A;Ripk3-/- mice (Figure 4) is very intriguing, however, the current analyses appear not to have enough statistical power to reach definitive conclusions. The authors should increase the N for both male and female mice for SOD1-G93A;Ripk3-/- mice. By doing so, it is hoped that the trend for more improved behavioral phenotypes in Figure 5 will become more obvious.

3. The histopathology analyses in Figure 6 showed no differences in MN numbers between SOD1-G93A and SOD1-G93A;Ripk3-/-. However, these analyses were performed at P140. Such analyses should be performed in at least two time points, at early symptomatic and end-stage.

4. The data in Figure 7 provide no additional information compared to what have been extensively published in the literature. The authors should perform the same analyses in SOD1-G93A;Ripk3-/- mice to show whether there is any change.

5. Finally, the results in Figure 8 are disconnected from the entire study. This reviewer is mystified by the inclusion of these results as they are completely irrelevant to the focus on Ripk3 in the SOD1-G93A model. This figure should be removed.

Reviewer #2

This is a well-designed and well-executed set of experiments designed to demonstrate the involvement of necroptosis, a form of programmed cell death, in ALS neurodegeneration by quantifying trasncripts and protein levels of core necroptotic genes in affected regions of the CNS in the SOD1 mouse model of ALS and validation in patient post-mortem tissue. In addition the authors sought to characterize the benefits of an anti-necroptosis strategy on motor performance and motor neuron survival in ALS mice to provide evidence to support this approach to treat ALS. The robust results obtained challenge the suggestion existing in the literature that targeting RIPK3 to abrogate necroptosis will provide therapeutic benefit in ALS.

The paper is well executed. the only concern that I would point out is for FIg1, which provide the entire premise to the conclusions of this study:

- the involvement of necroptosis genes and they relative protein levels in disease should be quantified against the control overexpressor human WT-SOD1 mouse model (FIG.1). This will provide the strongest evidence in support of upregulation of the necroptosis pathways in the ALS mouse model and will strengthen even more the conclusion of the pre-clinical studies. I believe this aspect is of paramount importance since the conclusions of this study challenge existing data in the literature.
